# EHMTI-0070. Effect of occlusal sprint in migraine patients

**DOI:** 10.1186/1129-2377-15-S1-G35

**Published:** 2014-09-18

**Authors:** M Takeuchi, J Saruta, M Kato, H Igarashi, T Kawata, K Tsukinoki

**Affiliations:** 1 Department of Oral Sciences Division of Orthodontics, Graduate School of Kanagawa Dental University, Yokosuka, Japan; 2 Department of Oral Sciences and Research Institute of Salivary Gland and Health Medicine, Graduate School of Kanagawa Dental University, Yokosuka, Japan; 3 Department of Internal Medicine, Fujitsu Clinic, Kawasaki, Japan

## Introduction

In recent years, research has progressed about the relevance of the Temporomandibular joint (TMJ) and headache, we also feels malocclusion are involved in the background. However, induction factors of migraine are many. In this study, we carried out splint therapy for migraine patients. We summarize the features of malocclusion and its effectiveness.

## Methods

The study population was composed of 14 migraine patients recruited at the Fujitsu clinic. All subjects were diagnosed with migraine by the second edition of the International Classification of Headache Disorders (mean age=40.1). For all subjects, we took impressions of the upper and lower teeth, face bow transfer and questionnaires about TMJ. On the dental model, we made occlusal sprint for patients and they used them for three months. We evaluated their change of migraine by MIDAS and HIT-6. Based on these data, the entire sample was divided into the following two groups. Group1: improvement of migraine.Group2: no change or worsened migraine. About two groups, we compared their dental models by the 3D digitizer. The statistical processing was used SPSS (Version 16.0 for Windows), Mann-Whitney U test.

## Results

There was a significant difference in Posterior occlusal Plane (POP) between group 1 and 2. POP of group 1 was significantly steeper than group 2(p=0.017).

## Conclusions

In migraine patients, there was a difference in the characteristics of the occlusion between the groups. It suggests the potential for improvement of migraine due to occlusion therapy. We want to continue further research from the point of view of dentist.

